# Low frequency of allergy referral for penicillin allergy evaluation in an urban Boston primary care setting

**DOI:** 10.1016/j.jacig.2022.09.004

**Published:** 2022-10-26

**Authors:** Alysse G. Wurcel, Rubeen Guardado, Christina Ortiz, Charles R. Bornmann, Joseph Gillis, Kristin Huang, Shira Doron, Maureen Campion, Kimberly G. Blumenthal

**Affiliations:** a Tufts University School of Medicine, Boston, Mass; b Department of Medicine, Division of Geographic Medicine and Infectious Diseases, Tufts Medical Center, Boston, Mass; c Department of Medicine, Division of General Internal Medicine, Tufts Medical Center, Boston, Mass; d Division of Rheumatology, Allergy and Immunology, Massachusetts General Hospital, Boston, Mass; e Harvard Medical School, Boston, Mass

**Keywords:** Penicillin allergy, disparities, delabel, β-lactam

## Abstract

**Background:**

A key strategy to combat the public health crisis of antimicrobial resistance is to use appropriate antibiotics, which is difficult in patients with a penicillin allergy label.

**Objective:**

Our aim was to investigate racial and ethnic differences related to penicillin allergy labeling and referral to allergy/immunology in primary care.

**Methods:**

This was a retrospective study of Tufts Medical Center’s Boston-based primary care patients in 2019. Univariable and multivariable logistic regression models were used to examine demographic associations with (1) penicillin allergy label and (2) allergist referral.

**Results:**

Of 21,918 primary care patients, 2,391 (11%) had a penicillin allergy label; of these, 249 (10%) had an allergist referral. In multivariable logistic regression models, older age (adjusted odds ratio [aOR] = 1.06 [95% CI = 1.04-1.09]) and female sex (aOR = 1.58 [95% CI = 1.44-1.74]) were associated with higher odds of penicillin allergy label carriage. Black race (aOR = 0.77 [95% CI = 0.69-0.87]) and Asian race (aOR = 0.47 [95% CI = 0.41-0.53]) were associated with lower odds of penicillin allergy label carriage. In multivariable regression, allergist referral was associated with female sex (aOR = 1.52 [95% CI = 1.10-2.10]) and Black race (aOR = 1.74 [95% CI = 1.25-2.45]). Of 93 patients (37%) who completed their allergy visit, 26 (28%) had received penicillin allergy evaluation or were scheduled to receive a penicillin allergy evaluation at a future visit.

**Conclusions:**

There were racial differences in penicillin allergy labeling and referral. Allergy referral for penicillin allergy assessment was rare. Larger studies are needed to assess penicillin allergy labeling and delabeling with an equity focus on optimizing patient health outcomes.

## Introduction

Antimicrobial resistance is a global public health crisis, further perpetuating racial and ethnic health disparities.^1^ People reporting penicillin allergy are at increased risk of antibiotic-resistant infections, mortality, and increased health care costs.^2^ Once a patient is labeled in the electronic medical record (EMR) as having a penicillin allergy, many clinicians avoid prescribing β-lactam antibiotics owing to concern regarding cross-reactivity, even though this concern is largely unwarranted.^3^^,^^4^ Removing an inaccurate penicillin allergy label from a patient’s EMR can improve clinical outcomes.^4^ Although 10% of people report having a penicillin allergy, up to 95% of people who report a penicillin allergy do not have a true IgE-mediated reaction and are able to tolerate penicillin.^5^^,^^6^ The American Academy of Allergy, Asthma & Immunology, the Infectious Diseases Society of America, the Society for Healthcare Epidemiology of America, and international allergy organizations recommend penicillin allergy delabeling (removal of inaccurate penicillin allergy labels).^7^

Prior studies considering race, ethnicity, and penicillin allergy demonstrate inconsistent findings. About three-quarters of participants in allergy registries are White, and prior studies suggest that White people are more likely to report a penicillin allergy.^8^ A study in New York City found that Asian people were less likely to report a penicillin allergy, but no difference was observed between Black people and White people.^9^ In a study of hospitalized patients with skin and soft-tissue infections, Black people were more likely to have a penicillin allergy label than White people.^10^ To date, racial and ethnic differences in allergist referral have not been assessed in an ambulatory population. In this study, we investigated demographic differences in penicillin allergy labeling and allergist referral in a large, urban primary care practice.

We identified patients with complete demographic detail who were seen in 2019 at Primary Care Boston, a single-site, academic practice at Tufts Medical Center in Boston, Massachusetts, that sees patients older than 18 years. Demographics, EMR-documented allergies, and allergy/immunology referrals were collected. Referral data were pulled from orders placed by the clinicians to allergists at Tufts Medical Center and surrounding practices. Race and ethnicity were from patient self-report at time of hospital registration. Race was categorized as White, Black, Asian, and other (Native American, Alaskan Native, Native Hawaiian, and Pacific Islander). Ethnicity was characterized as Hispanic/Latino or not. Penicillin allergy labels were determined by review of the EMR allergy list (review performed by C.R.B. and M.C.) by using coded and free-text entries entered in Logician (see the Supplementary Text in the Online Repository at www.jaci-global.org). Both active and inactivated (ie, resolved or delabeled) penicillin allergies were determined at the time of the 2019 primary care visit. If any penicillin was an active allergy in 2019, the patient was classified as having a penicillin allergy. The Tufts Health Sciences Institutional Review Board approved this study.

We report numbers with frequencies for binary variables and means with SDs for continuous variables. We performed univariable and multivariable analyses using logistic regression models for the outcomes of (1) penicillin allergy labeling and (2) allergy/immunology referral. Variables found to be statistically significant (*P* < .05) in the univariable analyses were included in the multivariable model. We reviewed records of patients with a penicillin allergy with a referral to allergy/immunology to assess whether they were evaluated by allergy/immunology and whether the penicillin allergy was discussed at the allergy visit. Analyses were performed using STATA 16 software (StatCorp, College Station, Tex).

## Results and discussion

Of 28,661 Primary Care Boston patients, 21,958 (77%) had complete demographic details and were included in the study (Table I). Patients had a mean age of 50 years (SD = 18 years), and 12,408 (57%) were female. The patients' racial distribution was White (63%), Asian (21%), Black (16%), and Other (<1%); 848 patients (4%) were of Hispanic ethnicity.

**Table I:** Characteristics of the primary care cohort (n = 21,958)

Characteristic	Value
Age (y), mean (SD)	50 (18)
Sex, no. (%)	
Male	9,550 (43)
Female	12,408 (57)
Race, no. (%)	
White	13,726 (63)
Black	3,587 (16)
Asian	4,605 (21)
Other	40 (<1)
Hispanic, no. (%)	848 (4)
Penicillin allergy label, no. (%)	2,393 (11)

There were 2391 patients (11%) with a penicillin allergy label who were of the White, Black, or Asian race and included in the logistic regression analysis. In multivariable regression, older age (aOR = 1.06 [95% CI = 1.04-1.09]) and female sex (aOR = 1.58 [95% CI = 1.44-1.74]) were associated with increased odds of penicillin allergy label carriage (Table II). Black race (aOR = 0.77 [95% CI = 0.69-0.87]) and Asian race (aOR = 0.47 [95% CI = 0.41-0.53]) were associated with lower odds of penicillin allergy label carriage.

**Table II:** Association between demographics and a penicillin allergy label

Characteristic	Penicillin allergy label (n = 21,918)∗		Univariable			Multivariable∗		
	No (n = 19,527)	Yes (n = 2,391)	OR	95% CI	*P* value	OR	95% CI	*P* value
Age (y), mean (SD)	50 (18)	53 (19)	1.11	(1.09-1.14)	< .0001	1.06	(1.04-1.09)	< .0001
Sex, no. (%)					< .0001			< .0001
Male	8,767 (92)	768 (8)	Ref			Ref		
Female	10,760 (87)	1,623 (13)	1.72	(1.57-1.88)		1.58	(1.44-1.74)	
Race, no. (%)					< .0001			< .0001
White	11,982 (87)	1,744 (13)	Ref			Ref		
Black	3,216 (90)	371 (10)	0.79	(0.70-0.89)		0.77	(0.69-0.87)	
Asian	4,329 (94)	276 (6)	0.44	(0.38-0.50)		0.47	(0.41-0.53)	
Hispanic, no. (%)					.08			—
No	18,756 (89)	2,314 (11)	Ref			—	—	
Yes	771 (91)	77 (9)	0.81	(0.64, 1.03)		—	—	

ORs compared penicillin allergy label with no penicillin allergy label.

*OR,* Odds ratio; *Ref,* reference.

∗ Multivariable models include sex, race, and number of allergy labels in the EMR. Some people were excluded because they were of a race other than White, Black, or Asian.

Among those patients with a penicillin allergy label, 249 (10%) were referred to allergy/immunology (236 of them [95%] were referred to the Tufts Allergy Group). In multivariable regression, allergy specialist referral was associated with female sex (aOR = 1.52 [95% CI = 1.10-2.10]) and Black race (aOR = 1.74 [95% CI = 1.25-2.45]) (Table III). In all, 1,313 patients without a penicillin allergy label (6.7%) were referred to allergy/immunology, with younger age, female sex, Hispanic ethnicity, and higher number of EMR-documented allergies associated with referral (see Table E1 in the Online Repository at www.jaci-global.org).

**Table III:** Association between demographics and allergy specialist referral among patients with a penicillin allergy label

Characteristic	Allergy referral (n = 2,391)		Univariable			Multivariable∗		
	No (n = 2,142)	Yes (n = 249)	OR	95% CI	*P* value	OR	95% CI	*P* value
Mean Age, (SD)	53 (19)	52 (17)	0.95	(0.89-1.02)	.171	—	—	—
Sex, no. (%)					<.001			.009
Male	715 (93)	53 (7)	Ref			Ref		
Female	1,427 (88)	196 (12)	1.85	(1.35-2.54)		1.52	(1.10-2.10)	
Race, no. (%)					.033			.013
White	1,582 (91)	162 (9)	Ref			Ref		
Black	315 (85)	56 (15)	1.74	(1.25-2.41)		1.74	(1.25-2.43)	
Asian	245 (89)	31 (11)	1.23	(0.82-1.86)		1.35	(0.89-2.05)	
Hispanic, no. (%)					.994			—
No	2,073 (90)	241 (10)	*Ref*	—		—	—	
Yes	69 (90)	8 (10)	1	(0.47-2.10)		—	—	

ORs compare allergy referral with no allergy referral.

*OR,* Odds ratio; *Ref,* reference.

∗ Multivariable models include sex, race, and number of allergy labels in the EMR.

**Fig 1:**
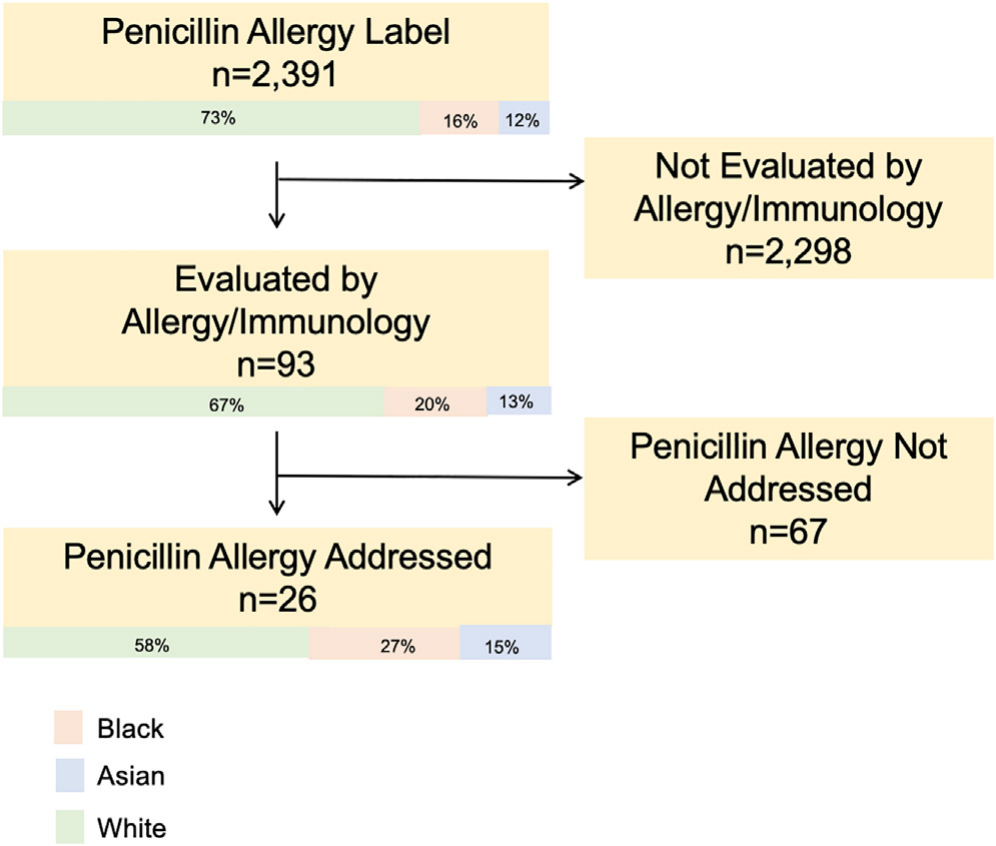
Penicillin allergy referral and evaluation for White, Black, and Asian patients in the Tufts Medical Center Primary Care practice.

A total of 93 patients (37%) completed their allergy/immunology visit (Fig 1), of whom 26 (28%) received a penicillin allergy evaluation or were scheduled to have an evaluation at a future visit. The 67 patients without a penicillin allergy discussion (72%) had documentation of other allergic concerns only (eg, seasonal allergies, food allergies). Allergy visits were completed with the same frequency regardless of whether the referral was to Tufts Allergy Group or a non-Tufts allergist (37% vs 38%).

In this Boston-based retrospective study of primary care patients, 11% of patients carried a penicillin allergy label. Black race and Asian race were associated with lower odds of penicillin allergy reporting than White race. Black race was associated with increased referral to allergy/immunology compared with White race. These racial differences in penicillin allergy reporting could be related to institutional and/or structural racism rather than to biologic differences.^11^ Increased antibiotic prescriptions to White children versus to Black, Asian, and Hispanic children leads to more opportunities to be labeled as having penicillin allergy.^12^, ^13^, ^14^ Racial differences may be related to clinicians’ limited ability to identify a rash on darker-skinned individuals, as dermatologic books have historically displayed drug-related rash on lighter skin tones.^15^, ^16^ A recent study demonstrated an association between HLA-DRB1∗10:01 and specialist-confirmed aminopenicillin allergy.^17^ Given a carriage rate of HLA-DRB1∗10:01 that is overrepresented in African ancestry versus in European ancestry by 2:1, it is important to recognize that we still do not know how race and ethnicity are associated with true penicillin allergy, given pervasive penicillin allergy mislabeling. Black race was associated with increased allergist referrals and will require further investigation, as this may be unrelated to penicillin allergy and instead explained by referrals for other atopic conditions, such as asthma, that are more prevalent and/or severe in Black patients.^18^

Tufts Medical Center is in the Chinatown neighborhood of Boston, as reflected in the large proportion of Asian patients in this study (21%). We uniquely demonstrated that Asian race was associated with lower odds of carrying a penicillin allergy label than White race. In the 1970s, China established routine penicillin allergy testing, even in patients who were penicillin-naive.^19^ Following improvements in US-China diplomatic relations, a growing number of people moved from China to Boston in the late 1970s and early 1980s.^20^ Although this practice of penicillin allergy testing in penicillin-naive individuals does not continue today,^19^ systematic penicillin allergy testing in China may be a reason why Asian people were less likely to carry a penicillin allergy label. Given Tufts Medical Center's location and existing academic-community partnerships,^21^ larger studies examining penicillin allergy reporting and delabeling in the Chinese population could be elucidating.

Most people with a penicillin allergy label who were evaluated by an allergist did not have any documentation of penicillin allergy discussion at visit. This is despite recommendations by allergy professional societies, including the American Academy for Allergy, Asthma & Immunology.^7^ Boston has a high density of allergists, and as such, the experiences of patients in this city likely underestimate the scope of the problem elsewhere.^22^ Allergists' routine incorporation of penicillin allergy evaluation into their discussions regardless of chief complaint is necessary. Delabeling incorrect penicillin allergies also needs to reach beyond allergist referral, as there are more than 30 million adults in the United States who have been incorrectly labeled as having a penicillin allergy but fewer than 6000 practicing allergists. Novel care models with nonallergist health care team members performing allergy histories, skin testing, and/or oral challenge are urgently needed as companions to ambulatory antibiotic stewardship efforts.^23^

Limitations of this study include its single practice site in a northeastern US city. Race and ethnicity data were complete in only three-fourths of patients. Race and ethnicity were administratively collected, and the process of asking patients their race and ethnicity can be problematic with administrative data sets.^24^ Although it is possible that patients sought an allergy specialist without a referral, this is unlikely given our insurance structures, and referrals both within and beyond Tufts were similarly captured. We did not collect data on or adjust for other socioeconomic indicators. Notably, most people living in Massachusetts have health insurance,^25^ and as such, insurance status is not usually a referral barrier. With so few patients referred specifically for penicillin allergy assessment, racial and ethnic differences in referrals for penicillin allergy assessments were not analyzed.

In this study, we observed racial differences in penicillin allergy labeling. There was an overall very low frequency of allergist referral for penicillin allergy assessment. Larger studies in primary care are needed to assess penicillin allergy labeling and delabeling associations with a health equity focus, particularly given the disparate impact of antimicrobial resistance on minoritized communities.

**Clinical implications**

There was a low frequency of allergist referral for penicillin allergy assessment in a primary care cohort. Penicillin allergy evaluation is not routinely considered in primary care.
