# Supplementary material for: Low frequency of allergy referral for penicillin allergy evaluation in an urban Boston primary care setting
Source: J Allergy Clin Immunol Glob. 2022 Oct 26;2(1):93–6. doi: 10.1016/j.jacig.2022.09.004 (PMC10509991; doi:10.1016/j.jacig.2022.09.004)
Supplement: Supplementary Materials [file mmc1.docx]

**Low Frequency of Allergy Referral for Penicillin Allergy Evaluation in an Urban Boston Primary Care Setting**

**Online Repository**

Tables (1)

Text (1)

| **Supplemental Table 1.** Association of demographics to allergy specialist referral among patients without penicillin allergy label. | | | | | | | | |
| --- | --- | --- | --- | --- | --- | --- | --- | --- |
| **Characteristic** | **Allergy Referral (N=19,527)** | | **Univariate** | | | **Multivariable** | | |
|  | **No (n=18,214)** | **Yes (n=1,313)** | **OR** | **95% CI** | **P-Value** | **OR** | **95% CI** | **P-Value** |
| Mean Age, (SD) | 50 (18) | 47 (17) | 0.91 | (0.88, .94) | <0.001 | 0.86 | (0.84, 0.90) | <0.001 |
|  |  |  |  |  |  |  |  |  |
| **Sex (%)** |  |  |  |  | <0.001 |  |  | <0.001 |
| Male | 8,359 (95) | 408 (5) | *Ref* |  |  | *Ref* |  |  |
| Female | 9,855 (92) | 905 (8) | 1.88 | (1.67, 2.12) |  | 1.64 | (1.45, 1.86) |  |
|  |  |  |  |  |  |  |  |  |
| **Race (%)** |  |  |  |  | 0.87 |  |  | - |
| White | 11,192 (93) | 790 (7) | *Ref* |  |  | *Ref* |  |  |
| Black | 2,973 (92) | 243 (8) | 1.16 | (.0.99, 1.34) |  | - | - |  |
| Asian | 4,049 (94) | 280 (6) | 0.98 | (0.85, 1.13) |  | - | - |  |
|  |  |  |  |  |  |  |  |  |
| **Hispanic (%)** |  |  |  |  | 0.01 |  |  | 0.01 |
| No | 17,514 (93) | 1,242 (7) | *Ref* | - |  | *Ref* | - |  |
| Yes | 700 (91) | 71 (9) | 1.43 | (1.11, 2.10) |  | 1.40 | (1.08, 1.80) |  |
|  |  |  |  |  |  |  |  |  |
| **Median Allergy Count, (Range)** | 0 (0 - 24) | 1 (0 - 29) | 1.28 | (1.24, 1.33) | <.0001 | 1.32 | (1.27, 1.37) | <.0001 |
|  |  |  |  |  |  |  |  |  |

| Supplemental Table 2: Average # of visit year to Primary Care Boston in 2019 | |
| --- | --- |
| **Race** | |
| American Indian | 2.7 |
| Asian | 2.7 |
| Black or African American | 2.94 |
| Native Hawaiian | 2.7 |
| White | 2.4 |
| **Ethnicity** | |
| Hispanic/Latino | 3.4 |
| Non-Hispanic/Non-Latino | 2.6 |
| **Gender** | |
| Female | 2.75 |
| Male | 2.54 |

Online Repository Text:

Allergy module allergens considered a penicillin allergy

Aminopenicillin

Amox

Amoxicillin

Amoxil

Amoxycillin

Ampicillin

Augmentin

Bicillin

Cillin

Cloxacillin

Dicloxacillin

Dicloxicillin

Dycill

Dynapen

Flucloxacillin

Moxatag

Nafcillin

Nallpen

Oxacillin

Pcn

Pencilin

Pencillin

Penicilin

Penicillin

Penicliin

Penicllin

Permapen

Pfizerpen

Piperacillin

Pipracil

Pivmecillinam

Pnc

Principen

Temocillin

Ticar

Ticarcillin

Timentin

Unacyn

Unasyn

Zosyn
